# Radiation-induced circulating microRNAs linked to echocardiography parameters after radiotherapy

**DOI:** 10.3389/fonc.2023.1150979

**Published:** 2023-05-18

**Authors:** Justyna Chałubińska-Fendler, Zuzanna Nowicka, Izabela Dróżdż, Łukasz Graczyk, Grzegorz Piotrowski, Bartłomiej Tomasik, Michał Spych, Jacek Fijuth, Anna Papis-Ubych, Piotr Kędzierawski, David Kozono, Wojciech Fendler

**Affiliations:** ^1^ Department of Radiotherapy, Military Institute of Medicine - National Research Institute, Warsaw, Poland; ^2^ Department of Biostatistics and Translational Medicine, Medical University of Łódź, Łódź, Poland; ^3^ Department of Clinical Genetics, Medical University of Łódź, Łódź, Poland; ^4^ Department of Radiation Oncology, Oncology Center of Radom, Radom, Poland; ^5^ Department of Teleradiotherapy, Regional Cancer Centre, Copernicus Memorial Hospital of Łódź, Łódź, Poland; ^6^ Cardiooncology Department, Medical University of Lodz, Łódź, Poland; ^7^ Cardiology Department, Nicolaus Copernicus Memorial Hospital, Łódź, Poland; ^8^ Department of Oncology and Radiotherapy, Faculty of Medicine, Medical University of Gdańsk, Gdańsk, Poland; ^9^ Department of Radiotherapy, Chair of Oncology, Medical University of Łódź, Łódź, Poland; ^10^ Department of Radiotherapy, Holy Cross Cancer Center, Kielce, Poland; ^11^ Department of Radiation Oncology, Dana-Farber Cancer Institute, Boston, MA, United States

**Keywords:** breast cancer, radiation-induced cardiac toxicity, echocardiography, biomarkers, microRNA

## Abstract

**Introduction:**

Patients treated with radiotherapy to the chest region are at risk of cardiac sequelae, however, identification of those with greatest risk of complications remains difficult. Here, we sought to determine whether short-term changes in circulating miRNA expression are related to measures of cardiac dysfunction in follow-up.

**Materials and methods:**

Two parallel patient cohorts were enrolled and followed up for 3 years after completion of RT to treat left-sided breast cancer. In the primary group (N=28) we used a a panel of 752 miRNAs to identify miRNAs associated with radiation and cardiac indices at follow up. In the second, independent cohort (N=56) we validated those candidate miRNAs with a targeted qPCR panel. In both cohorts. serum samples were collected before RT, 24h after the last dose and 1 month after RT; cardiac echocardiography was performed 2.5-3 year after RT.

**Results:**

Seven miRNAs in the primary group showed marked changes in serum miRNAs immediately after RT compared to baseline and associations with cardiopulmonary dose-volume histogram metrics. Among those miRNAs: miR-15b-5p, miR-22-3p, miR-424-5p and miR-451a were confirmed to show significant decrease of expression 24 hours post-RT in the validation cohort. Moreover, miR-29c, miR-451 and miR-424 were correlated with the end-diastolic diameter of the left ventricle, which was also confirmed in multivariable analysis adjusting for RT-associated factors.

**Conclusion:**

We identified a subset of circulating miRNAs predictive for cardiac function impairment in patients treated for left-sided breast cancer, although longer clinical observation could determine if these can be used to predict major clinical endpoints.

## Introduction

Breast cancer is the most common malignancy in women, accounting for 30% of all female cancers (1). Radiotherapy (RT) is a crucial element of treatment for breast cancer as it not only significantly decreases recurrence rates, but also has a prolongs patient survival (2, 3). Successful multidisciplinary treatment of breast cancer has resulted in a growing number of long-term survivors. Unfortunately, long-term survivors who underwent RT for breast cancer may have their risk of dying of cardiac disease increased 1.76-fold (4); this effect is further exacerbated by the concomitant use of cardiotoxic chemotherapy (5). This cardiac toxicity - both RT and chemotherapy associated - is a clinically relevant factor limiting quality of life and survival. Patients who have cancers of the left breast are particularly at risk of RT-associated cardiac toxicity (RACT). This is due to the anatomical location of the heart, that makes complete sparing of organs at risk (OARs), such as the heart and coronary vessels, impossible without compromising the radiation dose to the tumor bed. Even with the use of highly conformal RT and breath-hold techniques, the dose delivered to the OARs cannot be fully mitigated; however, the issue is especially relevant in low- and middle-income countries, where modern conformal RT techniques are not easily available (6). The risk of RACT thus necessitates further surveillance not only for recurrences but also impending cardiac dysfunction, which in turn necessitates the development of accurate techniques for prediction and management of treatment complications.

There is a variety of known risk factors of RACT, including presence or risk factors for coronary artery disease or other cardiovascular disease, the use of cardiotoxic chemotherapy, left-sided breast irradiation and earlier age at treatment (7), as well as individual RT planning and delivery techniques and dosimetric parameters (8, 9). Importantly, no minimum radiation dose threshold for risk exists and it is therefore recommended that the irradiated volume be minimized whenever possible (10). Still, very high heterogeneity exists between geographical locations and RT techniques with respect to mean heart doses (11, 12).

RACT is a serious and complex process, however cellular changes are not specific to RT. Ionizing radiation accelerates and aggravates damage due to oxidative stress, inflammation, and mitochondrial and endoplasmic reticulum injury (13). This damage leads to apoptosis and senescence of cardiomyocytes, smooth muscle cells, pacemaker cells, endothelial cells of cardiac vessels, and cells comprising the pericardium and cardiac valves.

Cardiomyocytes and pacemaker cells are particularly sensitive to oxidative stress as they have limited capacity to cope with free radicals (presumably due to low amounts of catalase) and high vulnerability to cell membrane oxidation as these cells possess huge amounts of phospholipids (14, 15). This higher sensitivity results in structural damage, cellular degeneration and dysfunction, reduced contractile ability, and ultimately myocardial remodelling. Cardiomyocytes have the capacity to divide, although much more slowly than endothelial cells. Irradiated endothelial cells become dysfunctional, with increased adhesiveness, permeability and premature senescence. Smooth muscle cells are triggered to proliferate and migrate (12). These processes ultimately lead to microvasculature damage, plaque formation and deposition of collagen and fibrin resulting in fibrosis.

It is upon development of fibrosis that symptoms and signs of cardiac toxicity are observed. Fibrosis ultimately leads to impaired ventricular filling and valvular function, acceleration of atherosclerosis (16) and increased risk of arrhythmias (17). The impaired function of heart substructures resulting in RACT manifests within the first 5 years and up to 15 years after RT (7). However, subclinical or asymptomatic cardiac changes may occur even over the first weeks and months (18, 19), long before clinical manifestation of RACT. During this asymptomatic period, excessive and progressive fibrosis occurs. Although fibrosis is considered irreversible, some signs of heart dysfunction may be identified earlier by ultrasound or heart MRI. Early imaging may, in this situation, prompt interventions to prevent late symptoms before irreversible changes occur. Microscopic changes that have already occurred cannot, however, be undone. Therefore, there is a need of markers that could reveal RACT very early, before any macroscopic changes occur. The most easily accessible would be blood-based, but multifaceted pathogenesis and heterogenous presentation of RACT poses a significant diagnostic challenge, not only in imaging but also in laboratory tests (20). The issue may be even more complicated as patients irradiated due to breast cancer could have undergone cardiotoxic chemotherapy or had cardiovascular disease prior to RT or existing risk factors of its development. Those factors may further hinder efforts to diagnose if the cardiac damage is due to radiotherapy or not.

RACT is mediated by inflammatory cytokines, including Transforming Growth Factor (TGF)-β (21). Several biomarkers, including C-reactive protein (CRP), N-terminal pro-B–type natriuretic peptide (NT-proBNP) and cardiac troponin T (cTnT) have been proposed for RACT (22–25), but none of them are specific. Some markers currently under investigation include galectin-3, myeloperoxidase and differentiation factor-15 (26). More recently, we have shown that Lipopolysaccharide-Binding Protein (LBP) levels correlate with dose-volume histogram (DVH) parameters and diastolic cardiac function in patients treated for breast cancer using RT. LBP was also correlated with DVH parameters in other radiation therapy-associated toxicities, e.g., lung toxicity (27).

Besides classical biomarkers, blood levels of microRNAs (miRNAs) have been linked to radiation-induced toxicity of normal tissues (28, 29). MiRNAs are short, non-coding RNA molecules that regulate gene expression and are involved in the processes relevant for RACT (30–32). Circulating miRNAs levels are remarkably stable in blood, even long time after freezing (33), and are easy to measure, making them attractive biomarkers. In previous studies, miR-22 and miR-433 have been proposed as markers of cardiac hypertrophy and fibrosis (34, 35). However, little is known about miRNA regulation during and after RT, and whether the impact of RT on miRNA regulation is only temporary or permanent. Changes in miRNA levels could be of use in terms of prevention of RACT in individual patients.

Here, we aimed to evaluate the potential of serum miRNAs as early biomarkers of cardiac toxicity in two prospectively followed cohorts of patients with breast cancer.

## Materials and methods

The study was approved by the Bioethics Committee of the Medical University of Lodz, Poland (RNN/291/17/KE) on 5th September 2017. After obtaining approval we recruited women with left-sided breast cancer, consecutively admitted to the Radiotherapy Department between March 2017 and August 2017. All participants provided written informed consent for participation. Inclusion criteria were: histologically diagnosed breast cancer, planned radical treatment with highly conformal RT (3D or Volumetric Modulated Arc Therapy (VMAT) or Intensity Modulated Radiation Therapy (IMRT)) either alone or in combination with systemic treatment, baseline ejection fraction > 56%, age > 18 years and signed informed consent for the study. Patients treated with breast-conserving surgery or mastectomy were deemed eligible for the study. Exclusion criteria were: any cardiac disease at baseline or any advanced chronic disease (heart failure—III/IV NYHA, renal failure—eGFR<30 mL/min/1.73 m2, liver failure—C or D score in Child–Pugh classification). Total dose of radiation was 5000 cGy delivered in 200 cGy daily fractions over 5 days a week, with a boost to surgical bed up to total dose of 6000-6600 cGy in the BCS group. All patients after mastectomy with indications for RT underwent chest wall and axillary irradiation not including internal mammary nodes. No boost was performed for surgical scar. Target volumes were contoured according to RTOG guidelines. RT was planned in Eclipse (version 10) with AriaTM (version 10) software with pencil beam calculation algorithm. Treatment was performed on Varian iX Linacs.

All patients underwent adjuvant chemotherapy prior to RT, if indicated. Concomitant treatment with systemic therapies other than chemotherapy was allowed, including treatment with trastuzumab, tamoxifen or aromatase inhibitors. From eligible patients, three serum samples were collected: prior to starting RT, 24 hours after receiving the last fraction of RT, and one month after the last fraction of RT, during a routine visit. All patients were invited for a follow-up visit 2.5-3 years after the end of RT, during which echocardiographic examination was performed. The echocardiographic variables evaluated at follow-up were directed at cardiac function evaluation with a particular focus on incipient dilatory cardiac dysfunction. A detailed list and reference ranges for the quantified parameters is presented in **Supplementary Table 1**. This group was evaluated echocardiographically by an experienced cardiologist using a Philips iE33 ultrasound machine and S5-1 cardiac sector probe.

A validation group of patients was recruited between March 2018 and August 2019 in the Department of Radiation Oncology in Holy Cross Cancer Center in Kielce, following the same inclusion and exclusion criteria. This group was followed up for at least 2.5 years after completion of breast cancer treatment. Patients from the validation group had serum samples collected under the same protocol as in the primary group: at baseline, 24 hours after administering the last fraction of RT and 1 month after RT. At follow-up, the patients were evaluated echocardiographically by an experienced cardiologist using Philips iE33 ultrasound machine (manufactured in 2010) and S5-1 cardiac sector probe. The sample size was estimated to be twice that of the primary cohort. MiRNAs associated with radiation exposure (significant differences between the second and other timepoints) and relevant reference miRNAs, described below, were selected for testing in the validation group.

Clinical characteristics of the study group and dosimetric data for the lungs and the heart are presented in **Table 1**. The patients from both groups did not differ in terms of age distribution and staging data. Several differences were noted in the strategy of RT planning, resulting in significant differences in several DVH parameters.

**Table 1:** Clinical characteristics of the study group.

Variable	Primary group	Validation group
	N [%] or mean ± SD	N [%] or mean ± SD
T: 1 2 3	16 [57%] 10 [36%] 2 [9%]	32 [57%] 16 [29%) 8 [14%]
N: 0 >0	15 [54%] 13 [46%]	35 [63%] 21 [37%]
Adjuvant chemotherapy: Yes No	11 [39%] 17 [61%]	26 [46%] 30 [52%]
Lymphadenectomy: Yes No	13 [46%] 15 [54%]	18 [32%] 38 [68%]
Type of surgery: BCS Mastectomy	21 [75%] 7 [25%]	41 [73%] 15 [27%]
Age [years]	57.94 ± 9.03	60.45 ± 7.98
Mean lung dose [Gy]	7.01 ± 4.63	8.77 ± 1.90
Mean lung V5	25.03 ± 18.23	57.34 ± 16.44*
Mean lung V20	11.94 ± 9.06	11.14 ± 3.12
Mean cardiac dose [Gy]	5.76 ± 3.83	9.87 ± 2.33*
Mean LAD dose [Gy]	22.31 ± 12.56	19.28 ± 9.45
Mean left ventricle dose [Gy]	7.50 ± 4.46	10.93 ± 2.90*
Mean cardiac V10	13.72 ± 14.79	36.28 ± 15.99*
Mean cardiac V30	5.18 ± 4.70	2.53 ± 2.46*

* P<0.05 for group comparison.

At all abovementioned timepoints, 2 ml of venous blood were collected to clotting activator-coated tubes (Beckton-Dickinson, Franklin Lakes, NJ, USA) and processed as in prior studies (28, 36). Briefly, the previously isolated and frozen serum was thawed on ice and centrifuged at 3000 x g for 5 min in a 4°C microcentrifuge. An aliquot of 200 μL per sample was transferred to a FluidX tube and 60 μl of Lysis solution BF containing 1 μg carrier-RNA per 60 μl Lysis solution BF and RNA spike-in template mixture was added to the sample, mixed for 1 min and incubated for 7 min at room temperature, followed by the addition of 20 μL Protein Precipitation solution BF. Total RNA was extracted from the samples using the miRCURY RNA isolation kit – Biofluids; high-throughput bead-based protocol v.1 (Exiqon, Vedbaek, Denmark) in an automated 96 well format. The purified total RNA was eluted in a final volume of 50 μl. The RNA was stored in a -80°C freezer. 19 μl RNA was reverse transcribed in 95 μl reactions using the miRCURY LNA™ Universal RT microRNA PCR, Polyadenylation and cDNA synthesis kit (Exiqon/Qiagen). cDNA was diluted 50 x and assayed in 10 μl PCR reactions according to the protocol for miRCURY LNA™ Universal RT microRNA PCR; each miRNA was assayed once by quantitative polymerase chain reaction (qPCR) on the microRNA Ready-to-Use PCR, Human panel I+II using ExiLENT SYBR^®^ Green master mix (Exiqon/Qiagen). Negative controls excluding template from the reverse transcription reaction was performed and profiled like the samples. In the validation cohort custom pick-and-mix arrays were designed (Exiqon/Qiagen) for miRNAs chosen for this part of the study and all miRNAs were quantified in triplicate. Amplification was performed in a LightCycler^®^ 480 Real-Time PCR System (Roche) in 384 well plates. The amplification curves were analyzed using the Roche LC software, both for determination of Cq (by the 2nd derivative method) and for melting curve analysis. Raw data from qPCR panel profiling were uploaded as **Supplementary Table 2**, as was normalized data processed as described in the Methods, as **Supplementary Table 3**.

### Data analysis

The amplification efficiency was calculated using algorithms similar to those of the LinReg software. All assays were inspected for distinct melting curves and the Tm was checked to be within known specifications for the assay. Furthermore, assayed miRNAs must have been detected with ≥ 5 Cqs less than the negative control, and with Cq < 37 to be included in the data analysis. miRNAs that did not pass these criteria were omitted from any further analysis. Cq was calculated as the 2nd derivative. All data were normalized to the average of assayed miRNAs detected in all samples (average – assay Cq). NormiRazor software was used to identify the best combination of normalizer miRNAs for validation group analysis out of all miRNAs expressed in sample in the primary cohort (37). Missing data were not imputed. Pairwise comparisons were performed with paired t-test with Benjamini-Hochberg adjustment for multiple comparisons. Hierarchical clustering was used to represent miRNA patterns across all three timepoints. Correlation was performed using the Spearman correlation test. MiRNA overrepresentation analysis was performed using the miEAA tool (38). All tests were two-sided and p values lower than 0.05 were considered as statistically significant. Analysis was performed using STATISTICA 13.1 (TIBCO Software, Palo Alto, CA, United States).

## Results

### miRNA profiles in sera of patients with breast cancer are influenced by RT

**Figure 1:**
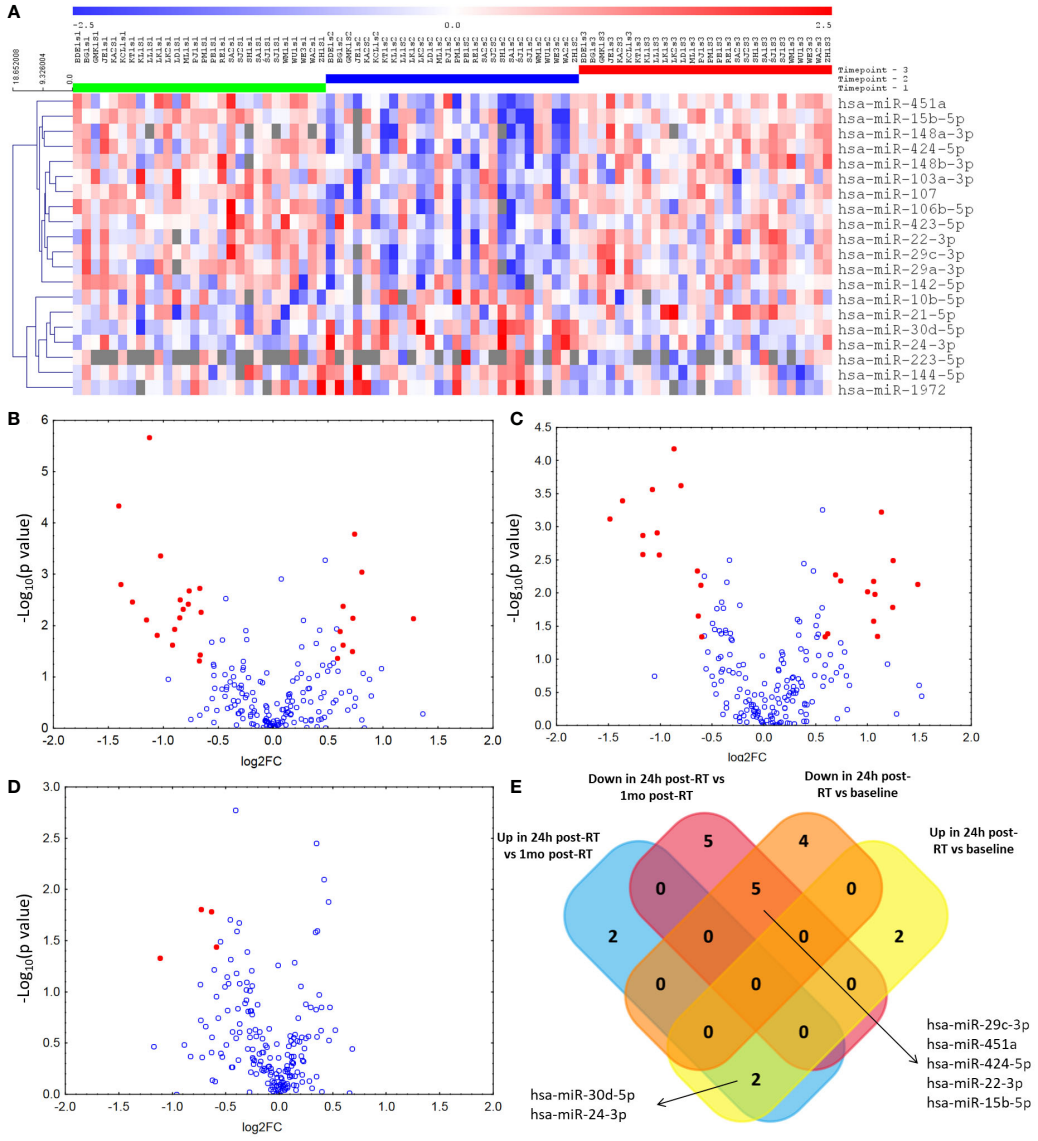
Results of miRNA expression analysis in the primary group. **(A)** Impact of radiotherapy for breast cancer on serum microRNA levels at three timepoints (1 – before first fraction of RT, 2 – 24 hours after the last RT fraction, 3 – 1 month after the last RT fraction) with at least one significant difference (after adjustment for multiple hypothesis testing) between either of the three timepoints. Next we identified miRNAs with fold changes > 1.5 or < 0.67 and unadjusted p values < 0.05 (represented by red dots) in between-timepoint comparisons: **(B)**) Volcano plot of miRNAs differentially expressed between the pre- and 24-hour post-RT timepoints. **(C)** Volcano plot of miRNAs differentially expressed between the 24-hour post-RT and 1-month post-RT timepoints. **(D)**) Volcano plot of miRNAs differentially expressed between the pre- and 1-month post-RT timepoints. **(E)** Among miRNAs with significantly different expression between the 24h post-RT timepoint and the two other timepoints we identified ones with consistent patterns of change and represented them on a Venn diagram.

To identify candidate biomarker miRNAs, we evaluated, using qPCR profiling, serum miRNA expression in samples collected at baseline, 24 hours and 1 month after radiotherapy, in the primary patient group (**Figure 1A**) recruited in the Department of Teleradiotherapy of the Medical University of Lodz. As anticipated, RT exerted changes in circulating miRNA profiles (**Figure 1B**). In the 24 h post-RT samples 9 miRNAs showed an >1.5 fold increased expression above baseline levels (**Figure 1C**). Eighteen miRNAs had lower expression (FC < 0.67) at the 24 h post-RT timepoint than the pre-RT timepoints, while 13 were down-regulated compared to the 1 month post-RT quantification (**Figure 1D**). At 1-month post-RT, virtually all miRNAs that showed an acute increase in expression reverted to their original levels, apart from 4 showing sustained decreased levels (miR-144, miR-335, miR-532 and miR-1972).

After adjustment for multiple comparisons, the levels of 2 miRNAs, hsa-miR-30d and miR-24, were increased at the 24 h post-RT timepoint versus both pre- and 1-month post-RT timepoints, while 5 were downregulated: miR-15b, miR-22, miR-29c, miR-424 and miR-451a (**Figure 1E**). None of the miRNAs that showed differential expression between the pre-RT and 1-month post-RT timepoints reached significance after adjustment (**Supplementary Table 4**).

### Levels of miRNAs altered by radiation are correlated with radiation dose to lungs and heart and follow-up echocardiographic evaluations

**Figure 2:**
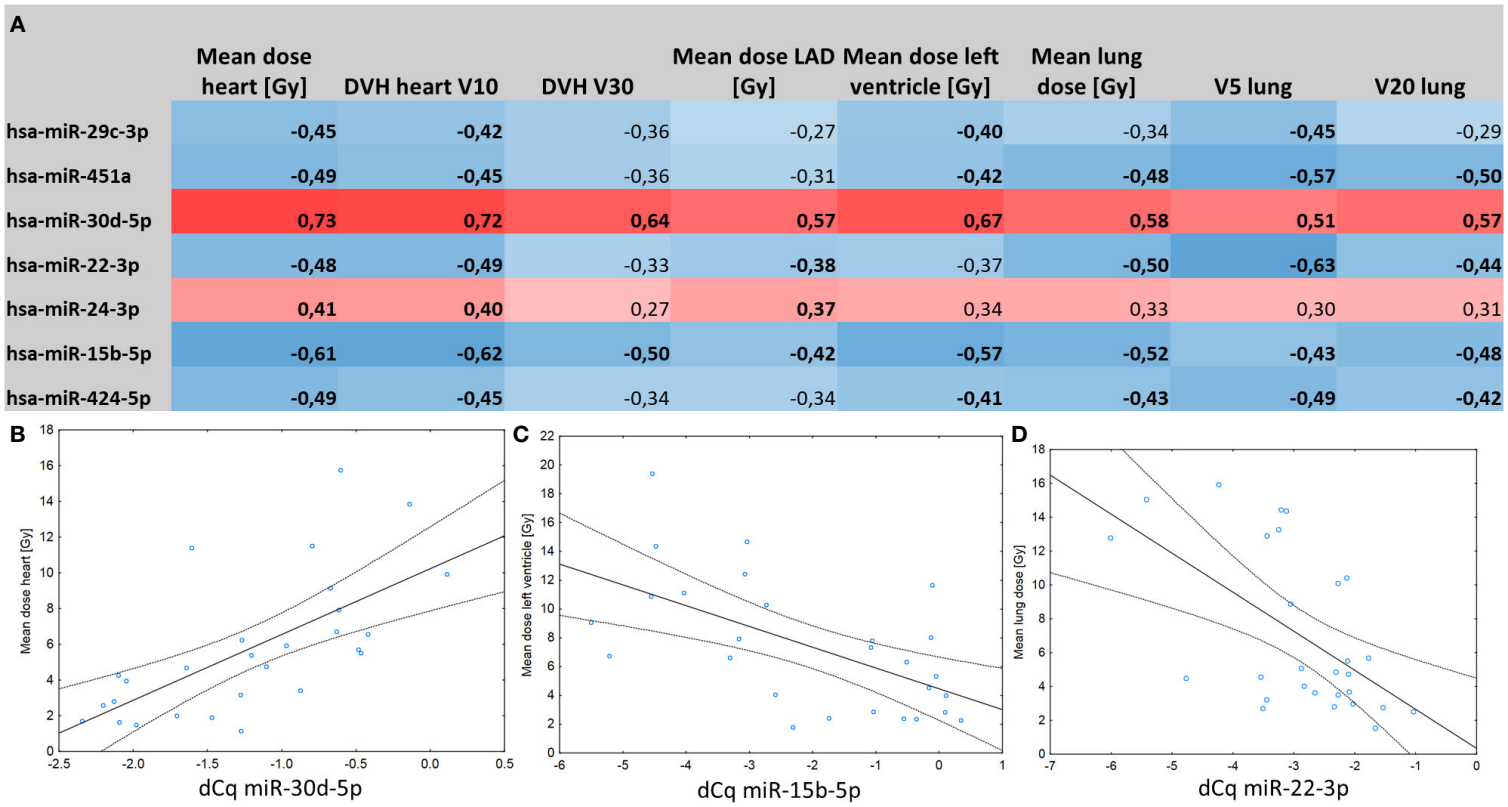
**(A)** Correlation matrix between post-RT miRNA expression levels and dose-volume histogram characteristics of the heart and lungs. Spearman correlation coefficients marked in bold are significant at p < 0.05. **(B)** Positive correlation between serum levels of miR-30d and mean dose delivered to the heart (r=0.73, p=0.01). **(C)** Negative correlation between serum levels of miR-15b and mean dose delivered to the left ventricle (r=-0.57; p=0.01). **(D)** Negative correlation between serum levels of miR-22 and mean lung dose (R=-0.50; p=0.02).

All of the abovementioned 7 miRNAs that showed significant transient increases or decreases at the 24 h post-RT timepoint were evaluated for correlation with heart and lung dose-volume histogram (DVH) parameters. miRNAs that showed significant increases after RT (miR-30d and miR-24) were positively correlated with DVH parameters (Spearman R ranging between 0.27 to 0.73), while miRNAs decreased after RT (miR-15b, miR-22, miR-29c, miR-424 and miR-451a) showed negative correlations with DVHs (Spearman R -0.27 to -0.63; **Figure 2**).

**Figure 3:**
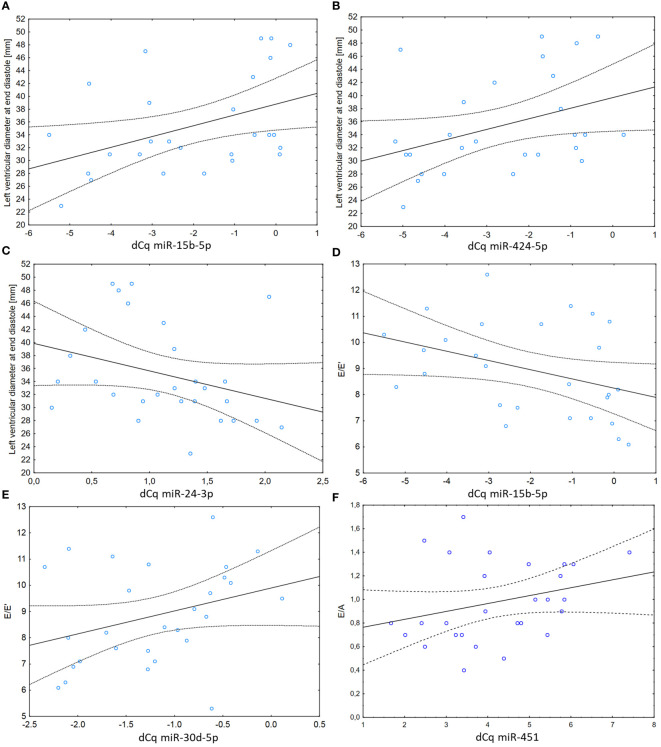
**(A)** Correlation between levels of miR-15b and thickness of the left ventricle wall in diastole at follow-up examination (r=0.42; p=0.03). **(B)** Correlation between levels of miR-424 and thickness of the left ventricle wall in diastole at follow-up examination (r=0.42; p=0.03). **(C)** Correlation between levels of miR-24 and thickness of the left ventricle wall in diastole at follow-up examination (r=-0.42; p=0.03). **(D)** Correlation between levels of miR-15b and the E/E’ ratio (an index that can be used to evaluate the LV filling pressure as defined in **Supplementary Table 1** – presented without units as a ratio of velocities) at follow-up examination (r=-0.41; p=0.03). **(E)** Correlation between levels of miR-30d and the E/E’ ratio at follow-up examination (r=0.35; p=0.06). **(F)** Correlation between levels of miR-451 and the E/A ratio (a marker of the function of the left ventricle of the heart as defined in **Supplementary Table 1** – presented without units as a ratio of velocities) at follow-up examination (r=0.32; p=0.09).

Associations between miRNA levels 24 h post-RT and echocardiographic variables of cardiac function assessed approximately 3 years after RT showed that miR-15b, miR-424 and miR-24 correlated with the end-diastole diameter of the left ventricle (r = 0.42; p = 0.03; **Figure 3A**, r = 0.42; p = 0.03; **Figure 3B** and r = -0.42; p = 0.03; **Figure 3C**), following the pattern of respective positive and negative associations with prior DVH parameters. Moreover, miR-15b and miR-30d correlated with E/E’, a measure of left ventricular filling pressure (r = -0.41; p = 0.03; **Figure 3D** and r = 0.35; p = 0.06; **Figure 3E**), while miR-451 correlated, although not significantly, with E/A ratio, a measure of left ventricular diastolic function (r = 0.32; p = 0.09, **Figure 3F**). Additionally, miR-29c (r = -0.49; p = 0.01) and miR-451 (r = -0.45; p = 0.02) negatively correlated with the Vmax through the tricuspid valve. None of the miRNAs correlated with the ejection fraction (all -0.2 < r < 0.2 and p > 0.15). Similarly, none of the dosimetric variables correlated significantly with either the left ventricle wall thickness in diastole or the E/E’ ratio. Means and standard deviations of the three echocardiographic variables were: E/e’ 8.84+/-1.85; E/A 0.98+/-0.33 and end-diastole diameter of the left ventricle 35.10+/-7.33 mm respectively.

Overall, these relationships encouraged us to select these miRNAs for further validation as candidate biomarkers capable of predicting RACT in patients undergoing RT for breast cancer. This selection was supported by the results of miRNA overrepresentation analysis across tissue and cell types, that showed 5 miRNAs to be overrepresented in fibroblasts (adjusted p = 0.009; miR-15b, miR-22, miR-24, miR-29c and miR-424). Among diseases, cardiomegaly was the most significant association (adjusted p < 0.001) with miR-15b, miR-22, miR-24, miR-29c, miR-30d and miR-451a being reported as involved in this disease. Moreover, significant (adjusted p = 0.011) association was noted for all 7 miRNAs with the “MicroRNAs in cardiomyocyte hypertrophy” pathway and 5 out of 7 in the “dilated cardiomyopathy” one (**Supplementary Table 5**).

### Validation of results by qPCR in an additional patient group

Fifty-six patients with left-sided breast cancer were recruited in the independent validation group prospectively recruited in the Department of Radiotherapy of the Holy Cross Cancer Center. Due to haemolysis and failure to collect two samples, 51 of the 56 patients had a complete set of 3 blood draws eligible for miRNA expression analysis. Candidate miRNA expression measured with qPCR was normalized to the average of miR-20a-5p, miR-23a-3p and miR-126-5p (top three-miR combination of normalizers selected using NormiRazor). Cardiac indices equaled: E/e’=10.03+/-2.70; E/A=0.90+/-0.28 and the Left ventricular end-diastolic diameter = 31.63+/-4.00 mm respectively.

**Figure 4:**
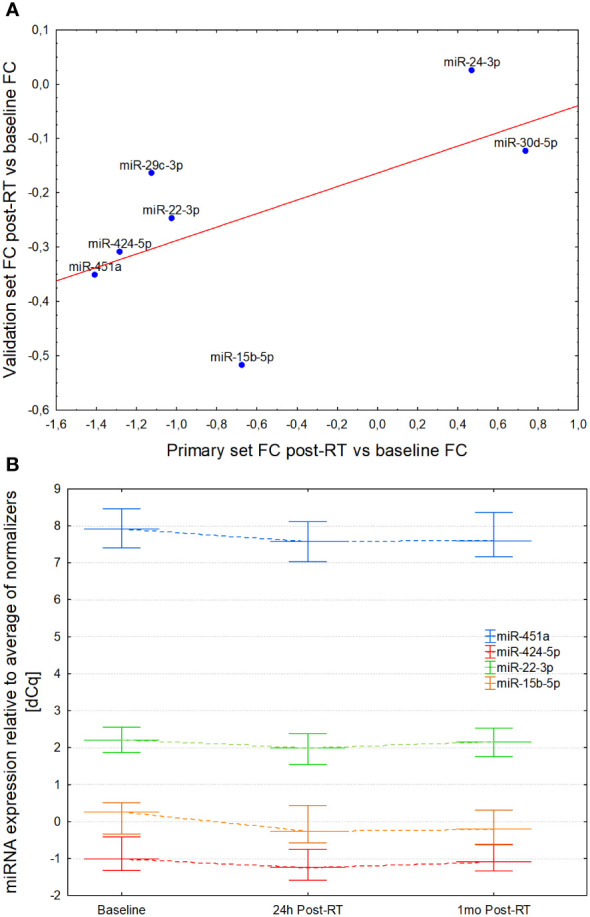
**(A)** Correlation of post-/pre-irradiation expression level fold changes in the primary and validation cohort. Both sets show similar pattern and magnitude of miRNA expression changes elicited by radiation exposure and subsequent normalization. **(B)** Serum expression levels of miR-451, miR-424a, miR-22-3p and miR-15b-5p at baseline, 24 hours and 1 month after completion of radiotherapy. In all cases the drop 24h post-RT was significant vs baseline values and reverted to values non-significantly different from the baseline at 1 month, with the exception of miR-15b-5p which remained decreased.

In this validation, the effects exerted by RT on the expression of 7 miRNAs were similar to those identified in the primary analysis, as demonstrated by correlation of fold changes between the post-RT and baseline timepoints in both groups (**Figure 4A**; R = 0.62; p = 0.140). Out of the 7 miRNAs, 4 (miR-15b-5p, miR-22-3p, miR-424-5p and miR-451a) showed significant RT-induced expression changes consistent with the primary dataset. Similar to the primary group, the differences observed for miR-22-3p, miR-424-5p and miR-451a post-RT disappeared 1 month after treatment (all p values > 0.15 except for miR-15b-5b, which remained significantly suppressed compared to baseline value; **Figure 4B** and **Supplementary Table 6**).

Three miRNAs measured immediately after RT in the validation group samples: miR-29c (R = 0.41; p = 0.03), miR-424 (R = 0.43; p = 0.024) and miR-451a (R = 0.49; p = 0.008) showed moderately strong correlations with left ventricle end-diastolic diameter. A non-significant correlation of the left ventricle end-diastolic diameter was observed with the mean heart dose (R = 0.26; p = 0.06), and that in turn was associated with DVH parameters and use of chemotherapy. In multivariable regression analysis performed to investigate independent association of miRNA levels with echocardiography indices, miR-29c (beta = 0.41; p = 0.03), miR-424 (beta = 0.39; p = 0.04) and miR-451 (beta = 0.43; p = 0.03) were significantly associated with left ventricle end-diastolic diameter. Correlations between other cardiovascular or dosimetric parameters with post-RT levels of circulating miRNAs were not significant (-0.15 < R < 0.15; p > 0.15).

## Discussion

Radiation-induced cardiotoxicity is a serious clinical problem in long-term breast cancer survivors that warrants efforts aimed at minimizing risk and identifying predisposed individuals. In this study, we found that several miRNAs measured in patient sera shortly (24 hours) after RT completion showed significantly altered expression, and these changes correlated with dosimetric parameters and echocardiographic findings performed 3 years after RT, pointing to their potential use as biomarkers. The radiation-induced changes in miRNA expression mostly reverted to normal 1 month after RT, reflecting the acute impact of radiation rather than ongoing physiological changes. That said, although the changes were transient, they may reflect acute processes that ultimately culminate in chronic pathophysiology in individuals prone to cardiac issues.

Previously, we reported that serum lipopolysaccharide-binding protein (LBP) concentrations changed dynamically over the course of RT in patients with left-sided breast cancer and are correlated with the E/E′ echocardiographic index reflective of diastolic function evaluated 3 years after the completion of RT. We therefore proposed LBP as an early biomarker of cardiac function (27). Since increased incidence of ischemic heart disease in women treated with RT for breast cancer is proportional to mean heart dose, with no apparent threshold (7), close monitoring of patients at risk of long-term cardiac sequelae is of great importance. Although limiting the dose of RT delivered to the heart by the use of conformal techniques and/or deep-inspiration breath-hold (39) is effective, these techniques may be insufficient for those at elevated cardiac risk. Furthermore, they are not widely available outside the highest-income countries. Reliance on solely dosimetric predictors does not account for biological heterogeneity of patients and individual susceptibility to radiation-induced toxicity. Current evidence suggests that personalizing RT based on the biological effect of radiation dose, predicted using tumor genomic characteristics (40, 41) may be an attractive strategy to improve patient outcomes. However, substantial interindividual variability in the susceptibility to radiation-induced myocardial damage (42, 43), underscores the importance of biological heterogeneity in this context, making reliance on a static, genomic index potentially inferior to a dynamically-changing biomarker.

Here, we explored the potential of serum miRNAs for early diagnosis of RT cardiotoxicity. We found that several miRNAs, including miR-22-3p, miR-24-3p, miR-29c-3p, miR-30d-5p, miR-424-5p, and miR-451a were significantly altered 24 hours post-RT and that their levels measured 24h post-RT were significantly correlated with dosimetric parameters.

MiR-30d, which was positively correlated with cardiac DVH parameters, has been reported to regulate cardiac remodeling in mouse models of ischemic heart failure (44); we and other authors have also previously identified miRNAs from the miR-30 family as radiation-inducible in mice, macaques and humans through an evolutionarily conserved regulatory mechanism (45). MiR-29c-3p expression decreased post-RT (46), consistent with previous reports regarding expression of the miR-29 family after radiation exposure; its expression was also inversely correlated with mean heart dose, heart V10 and with mean left ventricle dose. MiR-29c-3p has been extensively described in the context of pathological remodeling of the heart (47) and was shown to epigenetically regulate cellular senescence in fibroblasts and cardiomyocytes downstream of TGF-β signaling (48). Similarly, miR-22-3p expression decreased post-RT in both the primary and validation group, consistent with multiple earlier reports (46) and its expression was inversely correlated with cardiac dosimetry parameters. Serially measured miR-22-3p has been suggested to convey independent prognostic information for patients with chronic heart failure (49). miR-424, which was decreased post-RT and correlated with end-diastolic diameter of the left ventricle, has been reported to promote angiogenesis *in vitro* and in mice (50) and was proposed as a marker of disease progression in pulmonary hypertension (51), supporting its role in cardiac remodeling. miR-451a, which in our study was also decreased significantly immediately post-RT, is an established regulator of cardiac fibrosis and inflammation *via* pathways related to angiotensin II and matrix metalloproteinases 2 and 9 (52, 53).

Overall, miRNA over-representation analysis across tissue and cell types showed enrichment in fibroblasts and associations with cardiomegaly (miR-29c, -30d, -15b, -451a and -24) and cardiomyocyte hypertrophy. In general, left ventricle end-diastolic dimension is strongly associated with structural remodeling, contrary to the left ventricle end-systolic dimension and left ventricle ejection fraction (LVEF), which better represent systolic function than the structure of the left ventricle. In long term, radiotherapy may result in structural remodeling manifesting as left ventricular dilatation. Radiotherapy-induced long-term cardiotoxicity is associated with fibrosis, a process in which miRNAs may be involved. Therefore, the correlation between microRNA expression and LV end diastole dimension might be explained by the hypothetical role that the microRNAs play in the regulation of molecular mechanisms responsible for remodeling. While testing the tissue expression of these miRNAs in a clinical setting would be infeasible and mechanistic studies are required to identify the exact nature of these associations, these results serve as an additional line of evidence for the involvement of radiation-inducible miRNAs in processes that lead to long-term cardiac sequelae in patients with breast cancer treated with RT. Prior studies on radiation-induced toxicity in animal models identified several miRNAs associated with this process through experimental models. Notably, these include miR-223 (54), miR-21 (55) and miR-212 (56). Paper by Zhang et al. reported that miR-223 plays an important role in mitigating cardiac damage after irradiation. We observed an increase of its levels in the post-RT timepoint, but as the result was not statistically significant due to high interindividual differences in miR-223 expression patterns, this miRNA was not evaluated in the validation stage of the study. MiR-21 levels were upregulated significantly at the 1-month PostRT which is in line with this miR’s association with the fibrotic response. However, adjustment for multiple hypothesis testing negated this significance and we did not pursue this lead further. Within the studied sera we did not observe the expression of mir-212, which clearly does not undermine its importance in the cellular damage response, but precludes the use of its serum levels as a biomarker. Overall, two miRNAs identified through experimental works were partially confirmed by this study, despite the differences in the experimental setting, radiation doses delivered and tissue sampled. Finally, we revisited our prior work on radiation induced miRNA changes in patients treated for lung cancer (57). The dose distribution used in that study and sampling times were different but both miR-150 and miR-29a showed the same downregulation following exposure as previously, but were not among the top miRNAs evaluated in the validation set. All this amounts to support the strong association between radiation exposure and patterns of circulating miRNA expression, which may be a useful foundation for biomarkers of either exposure itself or, as shown in this work, its long-term sequelae.

Our study has several limitations. First, the correlations between miRNA expression and dosimetry parameters observed in the validation group were somewhat smaller than those in the primary group. A possible reason is the higher radiation dose delivered to the cardiac structures in the validation group, which was recruited at a different oncology centre and drawn from a potentially slightly different population of patients, possibly resulting in diminished differences in radiation-induced miRNA expression between patients (ceiling effect). Furthermore, due to sample size, the validation group was more heterogenous with respect to treatment strategy (mastectomy vs breast conservation therapy), resulting in variations in RT planning approaches. Nevertheless, the effects were generally validated in terms of direction of change and oftentimes significance, showing robustness of the findings. Thirdly, the strength of association of miRNAs with the evaluated effects was moderate (R~0.4) and no clinical endpoints were reached by the studied patients. However, the observed magnitude of effects was similar to that of dosimetric parameters which, given the independence of the identified miRNAs from DVH potentially allows for composite risk scores with better predictive performance. Due to the lack of cardiac outcomes in the observed patients, the analysis relied on association of miRNAs’ levels with echocardiographic parameters predictive for cardiac outcomes. The left ventricle end-diastolic diameter was correlated with radiation-associated miRNAs, but in all but one patient, remained within normal value range. This would suggest that a longer observation period would be needed to accumulate more data about the predictive capabilities of miRNAs towards clinically-evident cardiac sequelae. Finally, a different reason for the miRNAs dysregulation after treatment could be their association with breast cancer and the course of the disease rather than the impact of radiation itself. However, the miRNAs that we identified here as potential early biomarkers of cardiac toxicity were not reported among microRNAs significantly differentially expressed postoperatively in patients treated for breast cancer using surgery, but not radiotherapy (58). The miRNA expression changes were also correlated with radiation dose delivered to cardiac structures supporting their radiation-associated behavior rather than dependence on any residual tumor cells.

Taken together, the consistency of radiation-induced miRNA expression patterns between the two recruited patient cohorts and with prior studies shows that expression of circulating miRNAs as biomarkers in radiation oncology is a promising area for biomarker discovery for short- and long-term sequelae of RT.

## Conclusion

Significant RT-induced changes are observed transiently after the completion of treatment and those miRNAs are associated with dosimetric variables including doses to several cardiac structures. Considering also the links between specific miRNAs identified in this study and processes relevant for cardiac remodeling after radiotherapy, further large-scale should explore their potential to guide clinical management of patients with breast cancer at high risk of cardiac toxicity.

## Data availability statement

The expression profiling data using miRNA arrays were uploaded to the Gene Expression Omnibus database (https://www.ncbi.nlm.nih.gov/geo/query/acc.cgi?acc=GSE225243) under the accession number GSE225243.

## Ethics statement

The studies involving human participants were reviewed and approved by Bioethics Committee of the Medical University of Łódź, Poland (RNN/291/17/KE) on 5th September 2017. The patients/participants provided their written informed consent to participate in this study.

## Author contributions

JC-F and WF designed and planned the experiments. JC-F, ID, ŁG, GP, BT, MS, JF, AP-U and PK collected data and performed the experiments. WF and ZN performed statistical analyses. JC-F, ZN, WF, BT and DK interpret the results and wrote the manuscript. All authors contributed to the article and approved the submitted version. JC-F and ZN contributed equally.
